# Postbiotic Metabolite of *Lactiplantibacillus plantarum* PD18 against Periodontal Pathogens and Their Virulence Markers in Biofilm Formation

**DOI:** 10.3390/pharmaceutics15051419

**Published:** 2023-05-06

**Authors:** Widawal Butrungrod, Chaiyavat Chaiyasut, Netnapa Makhamrueang, Sartjin Peerajan, Wantida Chaiyana, Sasithorn Sirilun

**Affiliations:** 1 Department of Pharmaceutical Sciences, Faculty of Pharmacy, Chiang Mai University, Chiang Mai 50200, Thailand; widawal_b@cmu.ac.th (W.B.); chaiyavat@gmail.com (C.C.); netnapa.ma@cmu.ac.th (N.M.); wantida.chaiyana@cmu.ac.th (W.C.); 2 Innovation Center for Holistic Health, Nutraceuticals, and Cosmeceuticals, Faculty of Pharmacy, Chiang Mai University, Chiang Mai 50200, Thailand; 3 Health Innovation Institute, Chiang Mai 50200, Thailand; s.peerajan@gmail.com

**Keywords:** *Lactiplantibacillus plantarum*, postbiotic, probiotics, periodontal pathogens, virulence factors, biofilm formation

## Abstract

Alternative methods to reduce infectious diseases caused by bacterial pathogens and their virulence factors, biofilm formations, have arisen to reduce the pressure on existing or currently developed disinfectants and antimicrobial agents. The current strategies for reducing the severity of periodontal pathogen-caused disease by using beneficial bacteria and their metabolites are highly desirable. Probiotic strains of lactobacilli related to foods from Thai-fermented foods were selected and their postbiotic metabolites (PM) were isolated with inhibitory activity on periodontal pathogens and their biofilm formation. The PM from *Lactiplantibacillus plantarum* PD18 (PD18 PM) with the highest antagonistic effect against *Streptococcus mutans*, *Porphyromonas gingivalis*, *Tannerella forsythia* and *Prevotella loescheii* was selected from 139 *Lactobacillus* isolates. The minimal inhibitory concentration (MIC) and minimum biofilm inhibitory concentration (MBIC) values of PD18 PM against the pathogens ranged from 1:2 to 1:4. The PD18 PM demonstrated the ability to prevent the biofilm formation of *S. mutans* and *P. gingivalis* by showing a significant reduction in viable cells, high percentages of biofilm inhibition at 92.95 and 89.68%, and the highest effective contact times at 5 and 0.5 min, respectively. *L. plantarum* PD18 PM showed potential as a promising natural adjunctive agent to inhibit periodontal pathogens and their biofilms.

## 1. Introduction

Periodontal disease and dental caries are commonly found in the oral cavity. It has long been recognized that oral diseases, impacting between 20 to 50% of the world’s population, are the leading causes of tooth loss in both developing and industrialized countries [1]. Periodontal disease possesses a complicated etiology, causing inflammatory destruction of dentition’s supporting components and finally resulting in the loss of dentition [2]. The disease is caused by an imbalance between the host’s abnormal immunologic responses, personal susceptibility [2,3], and dysbiotic bacterial populations in the oral cavity, leading to the overgrowth of periodontal pathogens such as *Porphyromonas gingivalis*, *Tannerella forsythia* and *Prevotella loescheii* [4,5,6]. However, dental caries is caused by an imbalance between fermentable carbohydrates and cariogenic bacteria in dental plaque over time [7]. *Streptococcus mutans* is a dental caries starter strain [8] owing to its ability to produce exopolysaccharides (EPS) such as glucan and to rapidly form a mature biofilm on the tooth surface [9]. 

To survive and proliferate inside the host, these pathogens create a variety of virulent factors. *P. gingivalis*, one of the “red complex bacteria”, has several virulence factors including fimbriae, hemolysin, gingipains, capsules, outer membrane vesicles, lipopolysaccharides (LPS), and hemagglutinins [10,11,12]. The ability of fimbriae and capsules to attach to molecules on other bacteria as well as host tissues and cells facilitates the development of the biofilm. 

*P. gingivalis* applies virulence factors to escape immune system clearance, invades host cells, and takes advantage of host immune systems for colonization and survival, resulting in dysbiosis in the oral cavity, damage to tissue, and enhanced periodontitis [11,13,14]. Another dominant member of the “red complex” is *T. forsythia*, possessing virulence factors such as trypsin-like proteases, bacterial S-layer components, *Bacteroides* surface protein A (BspA), surface-lipoproteins, and hemagglutinin [15], which encourages the degradation of the periodontium, adhesion to gingival cell surfaces, the induction of cellular apoptosis, and a reduction in the immunological response in the host [15,16]. *P. loescheii* is a commensal microflora generally found on mucosal surfaces of the buccal cavity. It can switch to the oral pathogens caused by dysbiosis, affecting the host immune response and increasing the synthesis of various virulence factors including fimbriae, adhesin, hemolysin, proteases, enzymes such as nuclease, LPS, and EPS [17]. *S. mutans* is another microorganism correlated to periodontal disease. Its main virulence factors include acidogenicity, acid tolerance, and adhesion mechanisms enhancing biofilm formation, changing the biofilm’s physicochemical characteristics and accumulating *S. mutants* and other species in the oral cavity [18,19,20]. 

Biofilm formation is an important marker for increasing the severity of periodontal disease, which develops as dental plaque enhances the severity of antibiotic tolerance and treatment difficulties through its adhesion and biofilm development on medical devices, which can result in serious illness [21]. The traditional treatment of these diseases involves the mechanical removal of dental plaque, antibiotics, and chemical agents such as chlorhexidine [8,9,22,23,24]. These treatments may produce many side effects including pain, swelling, tooth sensitivity, drug resistance, and tooth discoloration [22,23,24,25]. Consequently, current strategies using naturally beneficial bacteria groups such as probiotics are highly desirable for balancing the oral bacterial community, reducing oral pathogens, and stimulating the host’s immune responses [7,23,26,27,28] that may decrease the severity of oral disease and prevent oral pathogen sustainably, which is similar to the immunomodulatory substance.

Probiotics are defined as live microorganisms which, when taken in appropriate quantities, have a positive impact on the health of the host [29]. Probiotics have several effective mechanisms such as increasing the amount of healthy microorganisms in the mouth, supporting oral environmental conditions, competing nutrients and adhesion areas with oral pathogens, secreting antimicrobial substances, and modulating immune responses [30]. Probiotics produce a wide range of advantages, but their use in products is restricted. The living cells of probiotics can still cause fermentation processes, which may alter the physicochemical properties and stability of the products. Furthermore, legal restrictions allow only the use of viable cells in certain products. 

To overcome such limitations, the beneficial substances produced by probiotics, called “postbiotics”, have been used as an alternative approach. Postbiotics were defined as metabolites, or non-viable bacterial products derived from probiotics that have biological activity in the host. Postbiotics are a new functional element because they successfully boost probiotics’ effectiveness without creating issues regarding survivability and colonization [27,30]. Therefore, postbiotics exhibit equal effectiveness as probiotics, constitute diversity in developing consumer products in various forms, and reduce the limitations of the use of living cells in products. Probiotics that will be a good source of postbiotics include lactobacilli or *Lactobacillus* (former name), and *Bifidobacterium*, which is a major group of probiotics, but this study was focused on *Lactobacillus* spp. because they are more easily cultured, safe for use, and widely implemented as a starter culture or probiotic bacteria in the food and beverage industry [31,32]. *Lactobacillus* spp. are widely found in various environments [33] and applied for use in various industries because the US Food and Drug Administration (US FDA) has classified them as Generally Recognized As Safe (GRAS) [9,32,34]. Postbiotic metabolites (PM) produced by *Lactobacillus* spp. include a variety of bioactive substances such as organic acids, hydrogen peroxide, bacteriocins, short-chain fatty acids, phenol, EPS, antimicrobial peptides, diacetyl, cofactors, immune-signaling substances, enzymes, vitamins, and secreted biosurfactants [9,35,36].

An important member of the genus *Lactobacillus* is the species *Lactiplantibacillus plantarum* (previously *Lactobacillus plantarum*) [37]. Related studies have reported that postbiotics produced by *L. plantarum* have antimicrobial activity against foodborne and spoilage bacteria consisting of *Listeria monocytogenes*, *Salmonella enterica*, *Salmonella* Typhimurium, *Escherichia coli*, and *Bacillus cereus* [32,38]. Their PM exhibit anti-biofilm activity against pathogens related to oral biofilm, including *P. gingivalis*, *Aggregatibacter actinomycetemcomitans*, *S. mutans*, *Streptococcus sobrinus*, and *Candida albicans* [39,40,41,42,43,44]. Some researchers reported that *L. plantarum* 299v decreased the levels of biofilms in the glass tube and saliva-coated hydroxyapatite produced by *S. mutans* [45,46]. 

The purpose of this present study was to investigate the effectiveness of postbiotic metabolites (PM) from selected probiotic lactobacilli strains against periodontal pathogens and bacteria correlated to periodontal disease and their biofilm formation.

## 2. Materials and Methods

### 2.1. Bacterial Strains and Growth Conditions 

The oral indicator bacteria included oral biofilm formation strains *S. mutans* ATCC 25175 and periodontal pathogens *P. gingivalis* ATCC 33277, *T. forsythia* ATCC 700191 and *P. loescheii* ATCC 15930, obtained from the Innovation Center for Holistic Health, Nutraceuticals and Cosmeceuticals, Faculty of Pharmacy, Chiang Mai University, Thailand. *S. mutans* was cultured in Tryptone Soya Broth (TSB) (Himedia, Mumbai, India) and incubated for 24 h at 37 °C and 5% CO_2_. *P. gingivalis* was cultured in TSB supplemented with yeast extract (Himedia, Mumbai, India), L-cysteine hydrochloride (Himedia, Mumbai, India), hemin (Sigma Aldrich, St. Louis, MO, USA), and vitamin K1 (United States Biological, Salem, MA, USA). *T. forsythia* was cultured in TSB supplemented with N-acetylmuramic acid (NAM) (United States Biological, Salem, MA, USA), and *P. loescheii* was cultured in modified chopped meat medium. The three periodontal pathogens were incubated in an anaerobic chamber (Bactron 300, Sheldon MFG. Inc., North Carolina, USA) with an atmosphere containing 5% H_2_, 5% CO_2_, and 90% N_2_ for 48 h (*P. gingivalis* and *P. loescheii*) or 5 days (*T. forsythia*). To produce biofilms, the indicator strains were grown in Brain Heart Infusion (BHI) broth (Himedia, Mumbai, India) supplemented with 2% sucrose (BHIS) (RCI Labscan, Bangkok, Thailand). BHI agar was used for the antimicrobial test. 

### 2.2. Lactobacilli Isolated from Fermented Foods

*Lactobacillus* sp. (former name) were isolated from 67 samples of fermented foods including fermented vegetables (pickled mustard greens, pickled cucumbers, pickled garlic bulbs, pickled mango, and pickled bamboo shoots), traditional fermented meat products (Nham (fermented ground pork sausage) and Sai-krok-prew (fermented pork sausage)), fermented fish products (Pla-ra and Pla-som), fermented soybean products (Tao-hoo-yee (fermented soybean curd)), and dairy products (cheese and yogurt). These were collected from local markets in Chiang Mai Province, Thailand. All samples were kept in sterile containers, transported to the laboratory, and stored at 4 °C until analysis. After that, 225 mL of phosphate-buffered saline (PBS) was used to homogenize 25 g of each sample. The mixture was transferred to sterile Petri dish, poured with de Man Rogosa Sharpe (MRS) agar (Himedia, Mumbai, India) containing 0.0125% (*w*/*v*) bromocresol purple (Fisher Scientific, Loughborough, UK), and incubated at 37 °C in microaerobic atmosphere with 10% CO_2_ for 24 to 48 h [4]. Colonies with yellow zone were selected and re-streaked in fresh MRS agar plates. Then, the isolates shown as rod shapes, non-spore-forming, Gram-positive, and catalase-negative (basically identified as lactobacilli group) were maintained in MRS broth with 15% (*v*/*v*) glycerol at −20 °C and working stocks were maintained at 4 °C. Before experiments were performed, these stocks were sub-cultured in MRS broth to obtain an active culture. 

### 2.3. Antimicrobial Activity of PM from Isolated Lactobacillus sp.

The antimicrobial activity of PM from isolated *Lactobacillus* sp. against *S. mutans*, *P. gingivalis*, *T. forsythia*, and *P. loescheii* was determined using the agar well diffusion assay. The isolated *Lactobacillus* sp. were activated in MRS broth and incubated at 37 °C for 72 h. *Lactobacillus* PM were collected by centrifugation and sterilized by filtration using a sterile syringe filter with a 0.22 µm pore size. The surfaces of agar plates were inoculated by spreading *S. mutans* over Tryptone Soya Agar (TSA) medium (Himedia, Mumbai, India) surface. *P. gingivalis*, *T. forsythia*, and *P. loescheii* were spread over the surface of supplemented TSA, NAM agar, and modified chopped meat agar, respectively. After setting the agar medium, a hole with a diameter of 5 mm was punched aseptically with a sterile cork borer and the agar wells were filled with the PM. Then, the plates were incubated based on growth conditions for each bacterium, as described above. A vernier caliper was used to measure the diameters of the inhibitory zone from the outer edge of the well to the outer edge of the clear zone. The PM from isolated *Lactobacillus* sp. showing antimicrobial activity against all indicator strains were selected for further study.

### 2.4. Characterizing and Determining Minimum Inhibitory Concentration (MIC) of Lactobacillus PM 

The types of antimicrobial substances in *Lactobacillus* PM were characterized using an adapted method from a related study [47]. The PM were adjusted to pH 7.0 ± 0.2 using 1 N NaOH to neutralize the acid inhibitory effects. For proteinaceous compounds, PM were treated with 1 mg/mL proteinase K (Sigma Aldrich, St. Louis, MO, USA) and catalase (Sigma Aldrich, St. Louis, MO, USA) was added to remove the hydrogen peroxide. The untreated PM (crude) served as control. The treated and untreated PM were sterilized by filtration using sterile membrane filters, and the minimum inhibitory concentration (MIC) test was conducted. The MIC assay was evaluated by microtiter broth dilution assay in 96-well plates [48]. *S. mutans*, *P. gingivalis*, *T. forsythia*, and *P. loescheii* were inoculated in broth medium and adjusted to 10^6^ CFU/mL, and then 100 µL of the different dilutions of treated and crude PM were added to the well containing 100 µL of each bacterial suspension and incubated under static conditions as described above. The MIC value was established as the lowest concentration of PM showing no turbidity.

### 2.5. Determining Minimum Biofilm Inhibitory Concentration (MBIC)

The experiment to study the potential of *Lactobacillus* PM on anti-biofilm activity was conducted on a 96-well flat-bottom plate. *S. mutans* and *P. gingivalis*, serving as representative oral biofilm formation strains, were diluted in BHIS to obtain the final concentration of 10^6^ CFU/mL. The diluted culture was added to the 96-well plate. The *S. mutans* plate was incubated at 37 °C, 5% CO_2_ for 24 h, and the *P. gingivalis* plate was incubated in an anaerobic atmosphere at 37 °C for 48 h in the presence and absence of serially diluted PM. The MBIC was defined as the lowest concentration of PM to inhibit biofilm formation [49].

### 2.6. Molecular Identification

The effective *Lactobacillus* sp. genomic DNA was isolated and purified for molecular identification using the Nucleospin^®^ DNA Kit according to the manufacturer’s instructions. The sequencing of the 16S rRNA gene was performed with the commercial services of Macrogen Inc. (Seoul, Republic of Korea) using the dideoxy chain termination method. The entire gene sequences were compared with those of the bacteria deposited in the Genbank nucleotide database (http://www.ncbi.nih.gov/, accessed on 18 May 2018) of the National Center for Biotechnology Information (NCBI) using the BLAST Program. The Neighbor-Joining technique was used to generate a phylogenetic tree. The Genbank nucleotide database’s (http://www.ncbi.nih.gov/, accessed on 18 May 2018) sequences were compared with those of effective *Lactobacillus* isolates.

### 2.7. Effect of Lactobacillus PM on Biofilm Formation and the Viability of Biofilm Cells 

The biofilms of *S. mutans* and *P. gingivalis* were cultured in BHIS and adjusted to 10^6^ CFU/mL. Then, 100 µL of each bacterial suspension was mixed with 100 µL of five various forms of selected PD18 PM and chlorhexidine gluconate (CHX) (Osoth Inter Laboratories, Chonburi, Thailand), including undiluted PD18 PM (PD18), diluted PD18 PM at dilution 1:2 (dPD18), and 0.12% CHX (CHX) serving as positive control. The combination of PM and CHX was used to evaluate the synergistic performance of both substances in antibiofilm activity, including PD18 PM combined with diluted CHX for 0.06% (PD18-dCHX) and diluted PD18 PM at dilution 1:2 combined with diluted CHX for 0.06% (dPD18-dCHX). The *S. mutans* plate was incubated at 37 °C, 5% CO_2_ for 24 h and the *P. gingivalis* plate was incubated in an anaerobic atmosphere at 37 °C for 48 h. The bacterial growth was measured by optical density (OD) at 595 nm using a microplate reader (SpectraMax M3, Molecular Devices, California, USA). To quantify the amount of formed biofilm, the samples were determined with crystal violet staining assay using spectrometric quantitation [50]. The planktonic cells and medium in wells were discarded and each well was gently rinsed three times with distilled water. The biofilm was stained with 0.4% crystal violet for 15 min, then gently rinsed three times with distilled water to remove the unbound dye and air-dried for 1 h. The stained biofilm was de-stained in 95% ethanol and the absorbance at 580 nm was measured using a microplate reader. The untreated biofilms of *S. mutans* and *P. gingivalis* served as negative control. The percentage of biofilm inhibition was calculated as follows [51]:

% biofilm inhibition = [(OD_negative control_ − OD_experiment_)/OD_negative control_] × 100; (1)

### 2.8. Effect of Contact Time of Lactobacillus PM on Oral Bacterial Biofilm

*S. mutans* and *P. gingivalis* biofilms were cultivated for 24 h using BHIS in 96-well flat-bottom plates. The formed biofilm was delicately washed with PBS before being exposed to five various forms of selected PD18 PM and CHX, as described above, by adding 100 µL of test solutions to the sample wells for 0.5, 1, and 5 min. To lessen the retained effect, the biofilm samples were rinsed with sterile Dey/Engley (D/E) Neutralizing Broth (Himedia, Mumbai, India), followed by PBS, and then allowed to dry. The biofilms were stained for 15 min at room temperature with 0.4% crystal violet and washed three times with PBS. The stained biofilms were redissolved with 95% ethanol and quantified in a microplate reader at 580 nm. The percentage of biofilm inhibition was calculated as described above.

### 2.9. CLSM Analysis of Bacterial Biofilm

The anti-biofilm activity of selected PM was investigated using confocal laser scanning microscopy (CLSM). *S. mutans* and *P. gingivalis* biofilms were formed on round glass cover slips (12 mm diameter) (Menzel-Glaser, Braunschweig, Germany) in a 24-well flat-bottom plate. The biofilm samples were cultured in BHIS, adjusted to 10^6^ CFU/mL, and incubated under the conditions described above. Then, the biofilms on the glass cover slips were subjected to PD18, PD18-dCHX, and CHX using the same procedure as described above for 5 min. The untreated biofilm served as a negative control. Afterwards, the biofilms were stained with SYTO-9 green fluorescent nucleic acid stain (Invitrogen, Life Technologies, Oregon, USA) for 15 min. A Nikon Laser Confocal Microscope C1 (Nikon Instruments, Tokyo, Japan) was used to examine the samples and observations were carried out with 20× and 40× lenses. The biofilm images were taken at 1024 × 1024 resolutions. EZ-C1 Version 3.90 was used to capture and analyze the images (Nikon software, Tokyo, Japan). 

### 2.10. Measuring Biofilm Formation and Residual Cells of Bacteria Using Scanning Electron Microscopy (SEM)

SEM was used to observe the bacterial biofilm’s morphology. Biofilm samples prepared on glass cover slips using the same procedure as described above were treated with 50 µL of test substances for 5 min, then gently rinsed with sterile D/E neutralizing broth and PBS, respectively. Biofilm samples were fixed with 2.5% glutaraldehyde at 4 °C overnight. Samples were dehydrated in ascending concentrations of ethanol (50, 70, 95, and 100%) for 15 min each. The dehydrated biofilm samples were critical-point dried, fixed on stubs, and sputter-coated with gold. The biofilm morphology was observed using SEM (Jeol JSM-6610LV, JEOL Ltd., Tokyo, Japan) on random areas of each sample using 1000× and 7000× magnifications. The bacterial morphology change was observed using 10,000× magnifications.

### 2.11. Evaluating the Capability of Reducing Biofilms from Typodont Teeth

The removal of biofilms method was performed using typodont teeth (Hexa Ceram Dental Laboratory, Chiang Mai, Thailand). The typodont teeth were sterilized and cultured in BHIS containing *S. mutans* and *P. gingivalis* in each sample, then incubated in static conditions to allow the biofilm to grow. The typodont teeth were rinsed with PD18 PM for 5 min, then gently rinsed with sterile D/E neutralizing broth and PBS, respectively. Then, 0.4% crystal violet was used to stain the treated typodont teeth for 15 min. After being washed three times with PBS, the biofilms on the surface of the typodont teeth were observed using a stereomicroscope (Stemi 508; Zeiss, Oberkochen, Germany) at 10× magnifications and evaluated using the ZEN 2 Software Program. The typodont tooth cultured in BHIS was used as a negative control, while the typodont tooth cultured in indicator stains without PD18 PM treatment was used as a positive control.

### 2.12. Statistical Analysis

Data from triplicate experiments were expressed as means and standard deviation of means. Statistical significance was determined using SPSS Version 17.0 of Windows (SPSS Inc., Chicago, IL, USA). One-way analysis of variance (ANOVA) was used to provide a statistical comparison. The outcomes were regarded as statistically significant at *p* < 0.05.

## 3. Results

### 3.1. Antimicrobial Activity of PM of Isolated Lactobacillus Strains

In total, 139 strains of *Lactobacillus* sp. were isolated from fermented food, comprising 51.29% of total lactic acid bacteria (LAB) isolates. The PM of all *Lactobacillus* isolates were examined for antimicrobial activity, which was expressed as the inhibition zone values (mm), as shown in Table 1. In total, 21 isolates (PD01-PD21) showed an inhibition zone against at least one of the tested strains. Only eight isolates were found to inhibit all indicator strains, including PD07, PD8, PD9, PD10, PD11, PD14, PD17, and PD18, and were selected for further studies. 

### 3.2. Characterizing and Determining the Minimum Inhibitory Concentration (MIC) of Lactobacillus PM 

The PM of eight selected *Lactobacillus* isolates, after being treated with proteinase K and catalase and neutralized, were used in the MIC test and the results are shown in Table 2 as a dilution titer. The crude and treated PM showed MIC values ranging from 1:2 to 1:4. The treated PM mostly lost antimicrobial activity against indicator strains, except for PD18. The crude and all treated PM of PD18 had antimicrobial activity against *P. loescheii* and showed MIC values ranging from 1:2 to 1:4. Moreover, the crude PD7 showed a strong inhibition against indicator strains, revealing MIC values of 1:4, for which PD7 and PD18 were chosen for further examination.

### 3.3. Determining Minimum Biofilm Inhibitory Concentration (MBIC)

Two *Lactobacillus* PM had the ability to inhibit biofilm formation from *S. mutans* and *P. gingivalis*. MBIC values are presented as a dilution titer of PM volume per total volume of reaction. The MBIC titers of PD7 were 1:2 for both *S. mutans* and *P. gingivalis*, while the MBIC values of PD18 were 1:2 and 1:4 for *S. mutans* and *P. gingivalis*, respectively. Although PD7 and PD18 metabolites showed the same MBIC values for *S. mutans*, PD18 had higher MBIC values than PD7 for *P. gingivalis* and exhibited antimicrobial activity against all indicator strains. Thus, PD18 PM was picked for the next assay.

### 3.4. Molecular Identification

**Figure 1:**
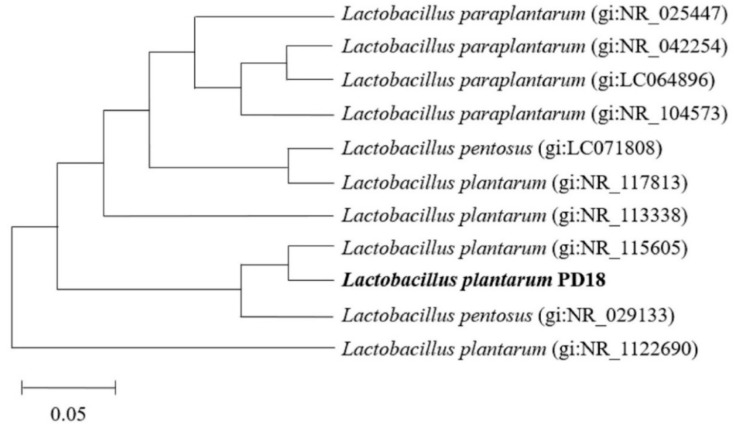
The phylogenetic tree of isolate PD18 was created by comparing the isolates’ 16S rRNA gene sequences to those in the database.

Based on the 16S rRNA analyses, GenBank alignment, a phylogenetic tree, was created based on the findings of the sequencing analysis of the 16S rRNA genes and is displayed in Figure 1. The PD18 showed 99% similarity with the sequence reported for *Lactiplantibacillus plantarum* (previously *Lactobacillus plantarum*) with accession number NR_115605.1 in the database.

### 3.5. Effect of Lactobacillus PM on Biofilm Formation and Viability of Biofilm Cells

**Figure 2:**
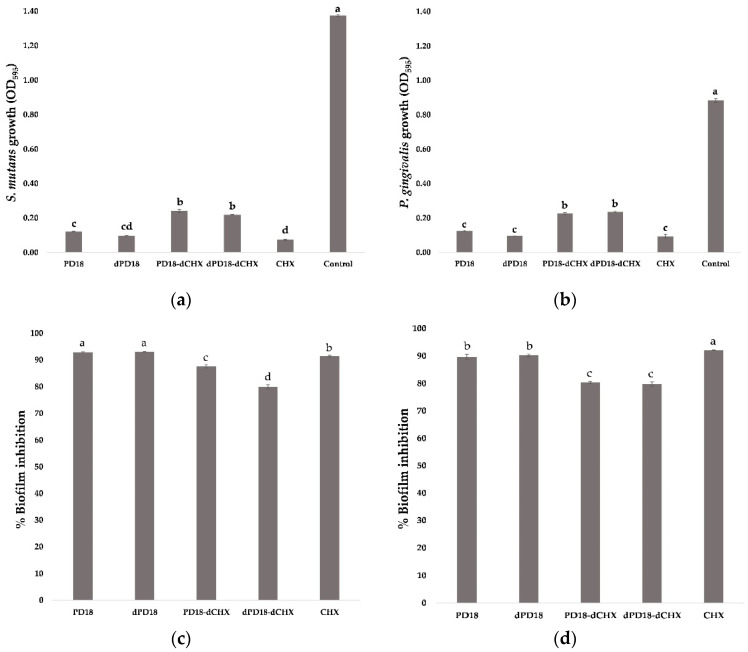
Effect of PD18 PM on bacterial growth and the percentage of biofilm inhibition for *S. mutans* (**a**,**c**) and *P. gingivalis* (**b**,**d**). The biofilm samples were treated with various forms of selected PD18 PM and CHX. Data are presented as means ± standard deviation of triplicate experiments. Significantly different (*p* < 0.05) values are represented by different letters above the bar. PD18 = PD18 postbiotic metabolites. dPD18 = diluted PD18 postbiotic metabolites at a dilution of 1:2. PD18-dCHX = PD18 postbiotic metabolites combined with diluted CHX for 0.06%. dPD18-dCHX = PD18 postbiotic metabolites at a dilution of 1:2 combined with diluted CHX for 0.06%. CHX = 0.12% chlorhexidine.

The effect of PD18 when co-cultured with *S. mutans* and *P. gingivalis* showed a significant downregulation of viable cells compared with controls, but no significant difference was noted in viable cells compared with CHX and dPD18 in both groups (Figure 2a,b). However, PD18-dCHX and dPD18-dCHX showed the highest viable cells, with no significant differences compared with other test substances. PD18 and dPD18 showed the highest percentage of biofilm inhibition for *S. mutans*, with no significant difference (92.95 and 93.09%), followed by CHX (91.46%), dPD18-dCHX (79.93%), and PD18-dCHX (87.63%), respectively. For *P. gingivalis*, CHX had the highest percentage of biofilm inhibition (92.11%), with significant differences from PD18 (89.68%) and dPD18 (90.24%), and the lowest percentages of biofilm inhibition were PD18-dCHX (80.30%) and dPD18-dCHX (79.76%) (Figure 2c,d).

### 3.6. Effect of Contact Time of Lactobacillus PM on Oral Bacterial Biofilm

**Figure 3:**
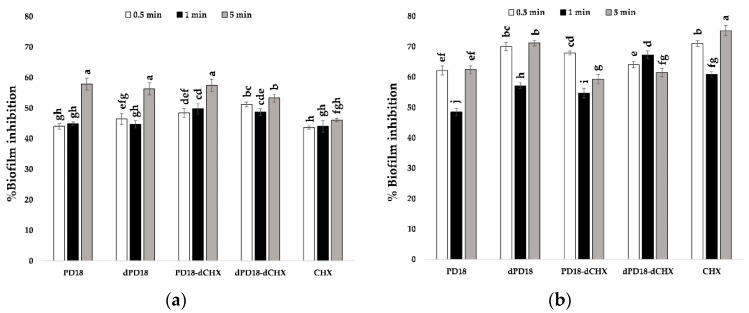
Effect of contact time of PD18 PM on the percentage of biofilm inhibition for *S. mutans* (**a**) and *P. gingivalis* (**b**). The biofilm samples were treated with various forms of selected PD18 PM and CHX for 0.5, 1, and 5 min. Data are presented as means ± standard deviation of triplicate experiments. Significantly different (*p* < 0.05) values are represented by different letters above the bars. PD18 = PD18 postbiotic metabolites. dPD18 = diluted PD18 postbiotic metabolites at a dilution of 1:2. PD18-dCHX = PD18 postbiotic metabolites combined with diluted CHX for 0.06%. dPD18-dCHX = PD18 postbiotic metabolites at a dilution of 1:2 combined with diluted CHX for 0.06%. CHX = 0.12% chlorhexidine.

When the test substances were exposed to the biofilm of *S. mutans* at 0.5, 1, and 5 min, the results showed in Figure 3 that PD18, dPD18, and PD18-dCHX at a contact time of 5 min had the highest percentages of biofilm inhibition, which were 57.90, 56.29, and 57.51%, respectively. This significantly differed from all metabolites at all time points, along with CHX, serving as a positive control. However, when CHX was tested against the *P. gingivalis* biofilm for 5 min, the percentage of biofilm inhibition (75.29%) was significantly higher among all metabolites at all time points, and PD18, at 1 min, was the lowest (48.50%).

### 3.7. Biofilm Observation by CLSM 

**Figure 4:**
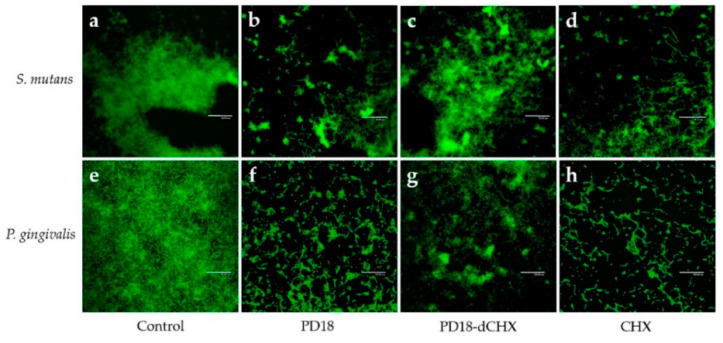
CLSM images of *S. mutans* and *P. gingivalis* biofilms treated with different test substances for 5 min. (**a**,**e**) *S. mutans* and *P. gingivalis* untreated biofilms served as control, (**b**,**f**) *S. mutans* and *P. gingivalis* biofilms treated with PD18, (**c**,**g**) PD18-dCHX, and (**d**,**h**) CHX. PD18 = PD18 postbiotic metabolites. PD18-dCHX = PD18 postbiotic metabolites combined with diluted CHX for 0.06%. CHX = 0.12% chlorhexidine.

The anti-biofilm activity of PD18 PM was analyzed using CLSM after staining for 5 min with SYTO-9 green fluorescent, which was used to indicate viable cells. The negative control (untreated biofilm) of *S. mutans* and *P. gingivalis* showed a thick biofilm, indicating much green fluorescence (Figure 4a,e). Reduced biofilm formation after treating with PD18 was found in both *S. mutans* and *P. gingivalis* (Figure 4b,f), which was similar to the results of CHX (Figure 4d,h), while PD18-dCHX also showed a dispersed and reduced density of biofilm (Figure 4c,g).

### 3.8. Morphologic Analysis Using SEM

**Figure 5:**
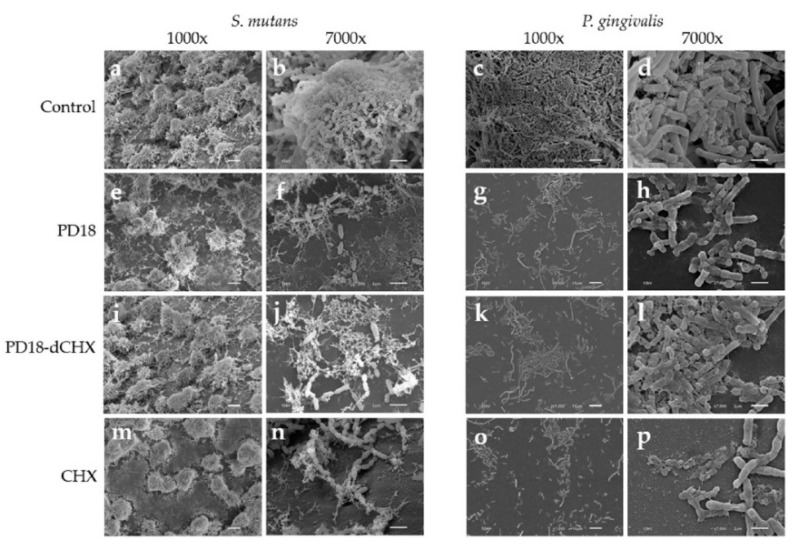
SEM images of *S. mutans* and *P. gingivalis* biofilm treated with different test substances for 5 min at 1000× and 7000× magnification. *S. mutans* untreated biofilms, which served as a control (**a**,**b**), *S. mutans* biofilms exposed to PD18 (**e**,**f**), PD18-dCHX (**i**,**j**) and CHX (**m**,**n**). *P. gingivalis* untreated biofilms served as a control (**c**,**d**), *P. gingivalis* biofilms exposed to PD18 (**g**,**h**), PD18-dCHX (**k**,**l**), and CHX (**o**,**p**). PD18 = PD18 postbiotic metabolites. PD18-dCHX = PD18 postbiotic metabolites combined with diluted CHX for 0.06%. CHX = 0.12% chlorhexidine.

The morphologies of *S. mutans* and *P. gingivalis* biofilms after exposure to various test substances for 5 min were observed using SEM (Figure 5). *S. mutans* and *P. gingivalis* untreated biofilms showed a high density of biofilms on glass cover slips (Figure 5a,c), revealing the intact morphology of the cells, which were cocci and rod shapes of *S. mutans* and *P. gingivalis* cells, respectively (Figure 5b,d). After treating with the test substances for 5 min, dispersed bacterial cells were observed. SEM images showed different surface structural details for PD18 and CHX-treated biofilm; the biofilm did not differ (Figure 5e–h,m–p).

**Figure 6:**
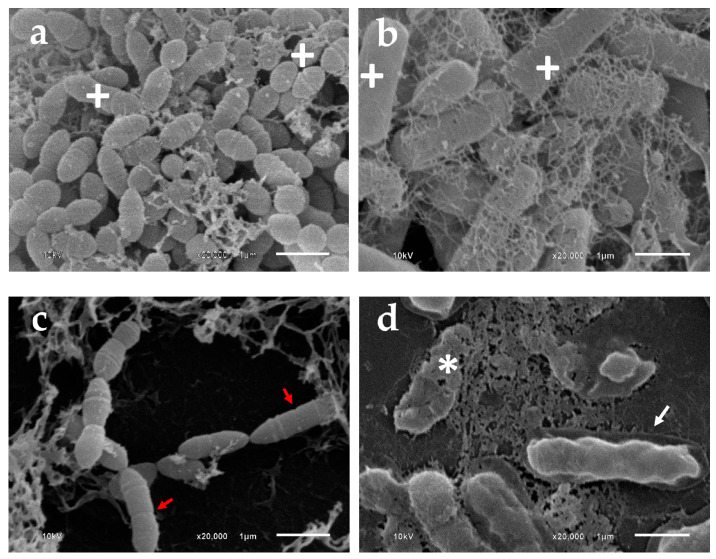
SEM images of oral bacterial biofilms untreated and treated with PD18 PM for 5 min at 20,000× magnification. (**a**) *S. mutans* and (**b**) *P. gingivalis* untreated biofilms served as control. (**c**) *S. mutans* and (**d**) *P. gingivalis* biofilm treated with PD18 PM. (+) intact morphology. (*) Cell membrane destruction and cytoplasm leakage. (White arrow) Shrinking dead cell. (Red arrow) Irregular shape [8].

High magnification SEM (Figure 6) showed a comparison of the morphology of *S. mutans* and *P. gingivalis* in the negative control and after treating with PD18. Both biofilm cells of the negative control exhibited regular bacterial morphology, with no obvious alterations to their physical components (Figure 6a,b), whereas PD18 displayed contrasting effects including an irregular shape for *S. mutans* (Figure 6c), shrinking dead cells, and cell membrane destruction, leading to cytoplasm leakage. Moreover, small particles were observed around the cell *P. gingivalis* (Figure 6d). The results indicated that PD18 PM was able to interrupt the bacterial biofilms of *S. mutans* and *P. gingivalis*.

### 3.9. Evaluating the Capability to Reduce Biofilms on Typodont Teeth

**Figure 7:**
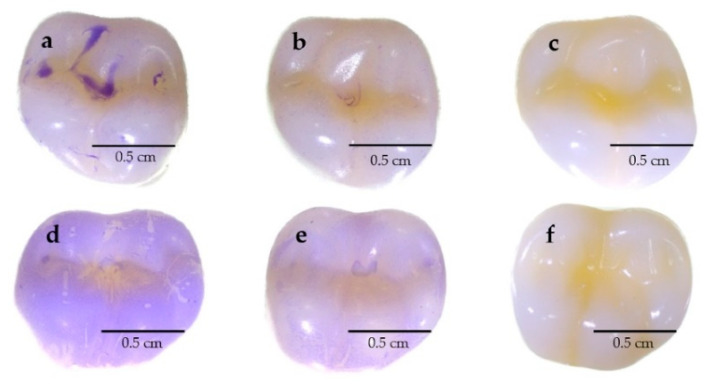
The oral bacterial biofilms on the surface of typodont teeth were treated and untreated with PD18 PM. (**a**) Untreated *S. mutans* biofilm (control). (**b**) *S. mutans* biofilm treated with PD18 PM. (**c**) Typodont tooth without oral bacterial biofilms and PD18 PM. (**d**) Untreated *P. gingivalis* biofilm (control). (**e**) *P. gingivalis* biofilm treated with PD18 PM. (**f**) Typodont tooth without oral bacterial biofilms and PD18 PM.

The ability to reduce the biofilm formation of *S. mutans* and *P. gingivalis* in PD18 PM was demonstrated on typodont teeth. After incubating typodont teeth with bacterial cells and PD18 PM, biofilm formation on typodont teeth was observed using a stereomicroscope. The PD18 PM showed reduced biofilm formation of both *S. mutans* and *P. gingivalis* compared with the negative control with no PD18 PM (Figure 7).

## 4. Discussion 

Periodontal disease is one of the most often reported chronic infectious dental disorders. Many dental diseases are normally treated with antibiotics and chemical agents, producing several adverse effects. The persistent use of antibiotics for dental diseases frequently results in multidrug-resistant bacteria, which can create long term negative effects on public health. Furthermore, the World Health Organization has considered antibiotic resistance as one of the top ten global health concerns to humanity [1]. In addition, some antibacterial drugs cannot not permeate into the bacterial biofilm formation. Compared with planktonic bacteria, bacterial biofilm is more resistant to antibiotics, which is the main cause of current bacterial drug resistance [52].

To overcome this issue, as potential techniques to control pathogen’s advancement in diseases, supportive therapies from diverse sources are being investigated. Creating probiotics for potential adjuvant therapy to control periodontal disease is one recommended technique [30]. Nevertheless, live microorganisms in probiotic formulations may lose the vitality of their cells. The challenges associated with production, storage, and distribution are exacerbated by the requirement to preserve cell viability. Moreover, using probiotics carries some risk that immunodeficient patients may experience negative outcomes. Thus, the use of postbiotics is receiving more attention because of their safety, efficiency, and stability, which are present during industrial operations and storage [53]. 

The PM of *Lactobacillus* sp. isolated from fermented foods were examined for their efficiency as postbiotics against periodontal pathogens including *P. gingivalis* ATCC 33277, *T. forsythia* ATCC 700191, *P. loescheii* ATCC 15930, and biofilm-associated oral pathogen, *S. mutans* ATCC 25175. Our results from the agar-well diffusion assay show that eight PM from the total of twenty-one PM of isolated *Lactobacillus* sp. exhibited antimicrobial activity against all indicator strains, including PD07, PD8, PD9, PD10, PD11, PD14, PD17, and PD18, accounting for 33.33% of selected isolates. Similar outcomes have been recorded. *L. plantarum* PD18 and its postbiotics showed antimicrobial activity against oral pathogens such as *S. mutans*, *A. actinomycetemcomitans*, *T. forsythia*, and *P. gingivalis* [4,40,45,46,54]. The interaction of competition and the release of antimicrobial compounds were proposed as mechanisms of inhibitory effects against oral pathogens such as organic acids, bacteriocin, hydrogen peroxide, secreted biosurfactants, and other antimicrobial substances [43,55]. Thus, the eight *Lactobacillus* PM were selected for further studies to characterize and determine the minimum inhibitory concentration (MIC).

The selected PM were investigated for the characterization of antimicrobial substances, e.g., proteinaceous compounds (such as bacteriocins), organic acids, and hydrogen peroxide, by treating them with proteinase K and catalase and neutralizing their pH. Proteinase K was used for proteinaceous compounds, catalase for hydrogen peroxide assay, and neutralized pH for organic acids assay. If the selected PM was treated with an assay and showed no effect, this indicated that the antibacterial effect came from that substance or in combination with other substances. Our results presented MIC titer values ranging from titer 1:2 to 1:4. Interestingly, all crude PM had antimicrobial activities, and most had 1:4 of titer that had a higher activity than treated PM, which may result from crude (natural extract) being a symbiosis of bioactive compounds. Moreover, the inhibitory action of all crude extract and treated PD18 PM did not disappear when tested with *P. loescheii*. This may have been caused by other postbiotics, except for the group of organic acids, hydrogen peroxide, and proteinaceous compounds such as lipase enzyme, biosurfactants, diacetyl, and extracellular metabolites, produced from *L. plantarum* as reported [35,39,43,46]. 

The sequencing analysis of the 16S rRNA genes reported that the PD18 had 99% similarity with *L. plantarum.* Several studies reported that *L. plantarum* is usually discovered in fermented food products. Mohd-Zubri et al. studied the cell-free supernatant of *L. plantarum* FT12 isolated from Malaysian fermented food [44]. In addition, *L. plantarum* RG11, RG14, RI11, UL4, TL1, and RS5 isolated from fermented food were also reported by Kareem et al. [56]. *L. plantarum* is still found in various fermented foods, as shown in previous studies such as those on fermented Sichuan sausages [57], sauerkraut, green olives, and cucumbers [58]. 

One of the key factors that might increase the severity of periodontal disease is biofilm formation. Difficulties in treating periodontal diseases are a result of their nature as a complex polymicrobial biofilm in dysbiosis [3]. These biofilms are difficult to remove through immunological responses [59]. Additionally, the biofilm produced by periodontal pathogens and associated bacteria including *S. mutants* and *P. gingivalis* decreases the effectiveness of antibiotic therapy [44,60]. *Streptococcus* species were the precursors of dental biofilms [61]. Patients with chronic periodontitis exhibited higher levels of *S. mutans* colonization in both saliva and subgingival plaque samples. Additionally, a favorable association was found with the periodontal parameters [59]. Moreover, the *S. mutans* count appeared to be directly correlated with worsening periodontitis severity among patients who were older and not receiving treatment [62]. *P. gingivalis* is considered to be the keystone pathogen in human periodontal disease [10,63]. *P. gingivalis* can create effective oxidative stress defense systems to survive when exposed to intracellular ROS, enhancing their aerotolerance ability [64,65], which increases the severity of this pathogen and its biofilms when they disperse to other sites of the host or contaminate medical devices. Therefore, these two bacterial strains were represented to evaluate the anti-biofilm activity of PD18 PM. The inhibition of biofilm formation might be associated with the antagonistic effect of LAB, the production of inhibitory substances, and the obstructive nature of the nutrients’ metabolism.

To study anti-biofilm activity, PM were co-cultured with *S. mutants*, and *P. gingivalis* and compared with CHX as a positive control. CHX was chosen as the antimicrobial agent in the present study because it is the most commonly used antibacterial agent in dentistry [66]. Due to its sustained broad-spectrum antibacterial action and plaque-inhibitory capability, CHX is regarded as the “gold standard” antiplaque in dental care products. However, even with the regular use of chlorhexidine, individuals may experience negative side effects and allergic responses. A bitter aftertaste may be experienced and can remain for several hours [66,67].

Our present study revealed that the selected PM had anti-biofilm activity against *S. mutans* and *P. gingivalis*, which significantly decreased their growth compared with the untreated control. Moreover, the percentage of biofilm inhibition of PD18 and dPD18 for *S. mutans* was significantly higher than that of CHX. The highest contact time of PD18, dPD18 and dPD18-dCHX increased the percentage of biofilm inhibition of *S. mutans* that significantly differed from that of CHX, while the highest percentage of biofilm inhibition of *P. gingivalis* was from CHX, which significantly differed from others. From the results, the antimicrobial and anti-biofilm activity of PD18 PM was found to be equivalent or better than CHX, as demonstrated by bacterial growth, the percentage of biofilm inhibition, and the effect of contact time on oral bacterial biofilm, which might decrease the use of CHX and its negative consequences.

The architecture and morphology of biofilms were evaluated using confocal laser scanning and scanning electron microscopy. After treating biofilms on glass cover slips with all test substances, they were stained with SYTO-9 and subjected to CLSM. The green color of SYTO-9 compared with the control that was not treated with any agent was significantly reduced. SYTO-9 green fluorescent labeling for nucleic acid within the polymeric matrix and inside living bacteria [50] correlated exactly with a cell’s viability. Hence, our results indicated that decreasing the SYTO-9 green treating with PD18, PD18-dCHX and CHX may cause pathogen cell abnormalities and death. To confirm this hypothesis, SEM analysis was performed. PD18 PM caused changes in the bacterial morphology (Figure 5c,d), and the antibacterial effect may have been the result of PD18 PM’s interaction with the bacterial membrane, eventually causing cell membrane destruction, cytoplasm leakage, osmotic imbalance, and cell death [68]. A similar result was reported by Yang et al. which exposed ClyR to *S. mutans* biofilms for 5 min and was observed using SEM. The outcome demonstrated that cells began to lyse, resulting in the production of ghost cells [8]. Additionally, CHX may change on the surface as a result of ionic interactions between the negatively charged EPS matrix and the positive CHX, leading to a rapid collapse of the surface-level matrix polysaccharides and loss in biovolume [66]. These results of SEM were consistent with determining the antimicrobial activity and CLSM assay, similar to related reports [66,69].

In addition to evaluating anti-biofilm activity co-cultured in microplates and on glass cover slips, we also exposed PM to biofilm on the surface of molar typodont teeth, which are in the area where the teeth cannot be brushed thoroughly. Food residue and organic matter can be found in the various niches, providing food for caries and periodontal pathogens that consequently form biofilms or plaque. This study was conducted on typodont using materials that have a surface similar to dentures for virtual use. Our results showed that PD18 PM was able to reduce the biofilm formation of *S. mutans* and *P. gingivalis*, which would assist in removing a significant portion of pathogenesis. The mechanism of anti-biofilm activity might be due to the secretion of postbiotics interfering with the formation of biofilm [9,44]. Srivastava et al. demonstrated that *L. plantarum* 108 PM have effective activity when reducing mixed biofilms of *S. mutans* and *Candida albicans*, which might be explained by the combined antibacterial peptides and other antimicrobial substances from *L. plantarum* 108, such as plantaricin and biosurfactants. They also found that the expression of all three *gtf* genes was significantly reduced by PM of *L. plantarum* 108, which consequently reduced the adhesion and biofilm production of *S. mutans*, according to several related studies [43,70,71]. Similarly, Moradi et al. reported that the biosurfactants from *L. plantarum* significantly decreased the amount of *Staphylococcus aureus* biofilm cells [72]. 

Based on the results above, *L. plantarum* PD18 PM could also be applied to oral care formulations such as mouthwash for preventing oral pathogens and their biofilms in further studies. In addition, this powerful postbiotic might be used in a variety of products in addition to those for oral hygiene, such as in antiseptics. 

## 5. Conclusions

This study obtained the efficiency postbiotic metabolite of *L. plantarum* PD18 isolated from fermented food that exhibited antimicrobial activity against *S. mutans*, *P. gingivalis*, *T. forsythia*, and *P. loescheii.* It also revealed anti-biofilm activity against two representative oral biofilm formation strains, *S. mutans*, and *P. gingivalis*, on both glass cover slips and molar typodont teeth. The results indicated that the PM of *L. plantarum* PD18 possessed the potential to be a promising natural adjunctive agent that could be applied in various oral care formulations to inhibit periodontal pathogens and their biofilms. However, the relevant studies have not indicated the main active substance and antimicrobial mechanisms involved. Further studies are needed to evaluate the composition of PD18 PM and mechanisms of anti-periodontitis pathogens in depth. A safety test will also be required before applying to products for human use. 

## Figures and Tables

**Table 1:** Antimicrobial activity of *Lactobacillus* PM against indicator strains.

*Lactobacillus* PM	Inhibition Zone (mm)			
	*P. gingivalis*	*T. forsythia*	*P. loescheii*	*S. mutans*
PD01	NI	7.58 ± 0.04 ^c^	NI	NI
PD02	NI	7.61 ± 0.11 ^c^	NI	NI
PD03	NI	7.04 ± 0.08 ^b^	NI	NI
PD04	NI	7.15 ± 0.06 ^b^	NI	NI
PD05	NI	7.10 ± 0.09 ^b^	NI	NI
PD06	NI	6.19 ± 0.21 ^a^	NI	NI
PD07	7.21 ± 0.10 ^c^	7.19 ± 0.12 ^b^	7.59 ± 0.06 ^e^	6.38 ± 0.04 ^b^
PD08	6.24 ± 0.12 ^a^	7.68 ± 0.24 ^c^	7.62 ± 0.15 ^e^	6.18 ± 0.06 ^c^
PD09	6.64 ± 0.12 ^b^	7.14 ± 0.14 ^b^	6.23 ± 0.06 ^b^	5.73 ± 0.10 ^d^
PD10	6.07 ± 0.05 ^a^	7.16 ± 0.28 ^b^	5.57 ± 0.12 ^a^	5.84 ± 0.16 ^d^
PD11	6.11 ± 0.07 ^a^	6.29 ± 0.17 ^a^	7.12 ± 0.09 ^d^	6.17 ± 0.06 ^c^
PD12	NI	NI	7.08 ± 0.14 ^d^	NI
PD13	NI	NI	7.14 ± 0.09 ^d^	NI
PD14	6.06 ± 0.06 ^a^	6.22 ± 0.16 ^a^	6.60 ± 0.13 ^c^	6.13 ± 0.11 ^c^
PD15	NI	NI	7.08 ± 0.20 ^d^	NI
PD16	NI	NI	7.18 ± 0.15 ^d^	NI
PD17	7.10 ± 0.16 ^c^	6.07 ± 0.27 ^a^	6.58 ± 0.08 ^c^	6.12 ± 0.07 ^c^
PD18	7.78 ± 0.19 ^d^	7.69 ± 0.11 ^c^	7.52 ± 0.07 ^e^	10.63 ± 0.05 ^a^
PD19	NI	NI	7.18 ± 0.04 ^d^	NI
PD20	6.17 ± 0.04 ^a^	NI	7.54 ± 0.10 ^e^	NI
PD21	NI	7.51 ± 0.11 ^c^	7.17 ± 0.10 ^d^	NI

Data are presented as means ± standard deviation of triplicate experiments. The different letters denote that the values are significantly different (*p* < 0.05) within an individual column. The inhibition zones were measured including diameter of agar well (5 mm). No inhibition zone (NI).

**Table 2:** The minimum inhibitory concentration (MIC) values of the crude and treated PM from *Lactobacillus* strains against indicator strains.

*Lactobacillus* PM	Treatment	*P. gingivalis*	*T. forsythia*	*P. loescheii*	*S. mutans*
PD7	Crude	1:4	1:4	1:4	1:4
	Neutralized pH	ND	ND	ND	ND
	Proteinase K	ND	1:2	ND	ND
	Catalase	ND	ND	ND	ND
PD8	Crude	1:2	1:4	1:4	1:2
	Neutralized pH	ND	1:4	1:2	ND
	Proteinase K	ND	ND	ND	ND
	Catalase	ND	ND	ND	ND
PD9	Crude	1:2	1:4	1:4	1:4
	Neutralized pH	ND	1:4	ND	ND
	Proteinase K	1:2	ND	ND	ND
	Catalase	ND	ND	1:2	ND
PD10	Crude	1:2	1:4	1:4	1:2
	Neutralized pH	ND	1:4	ND	ND
	Proteinase K	ND	ND	ND	ND
	Catalase	ND	ND	1:2	ND
PD11	Crude	1:2	1:4	1:4	1:2
	Neutralized pH	ND	1:4	ND	ND
	Proteinase K	ND	ND	ND	ND
	Catalase	ND	ND	1:2	ND
PD14	Crude	1:2	1:4	1:4	1:2
	Neutralized pH	ND	ND	ND	ND
	Proteinase K	ND	ND	ND	ND
	Catalase	ND	ND	1:2	ND
PD17	Crude	1:4	1:2	1:4	1:2
	Neutralized pH	ND	ND	ND	ND
	Proteinase K	ND	ND	ND	ND
	Catalase	ND	ND	ND	ND
PD18	Crude	1:2	1:4	1:4	1:2
	Neutralized pH	ND	1:2	1:2	ND
	Proteinase K	ND	ND	1:2	ND
	Catalase	ND	ND	1:2	ND

Data are presented as the dilution titer of cell-free supernatant volume per total volume of experiment in each well. Not detected (ND).

## Data Availability

The data presented in the manuscript is available on request from the corresponding author.
